# The Effect of Applying Treatment Sludge and Vermicompost to Soil on the Biodegradability of Poly(lactic acid) and Poly(3-Hydroxybutyrate)

**DOI:** 10.3390/polym17030352

**Published:** 2025-01-28

**Authors:** Seyma Nur Erkul, Selnur Ucaroglu

**Affiliations:** 1 Environmental Engineering Department, Faculty of Engineering, Bursa Uludag University, Bursa 16059, Turkey; seymakpinarr@gmail.com; 2 BUTEKOM Bursa Technology Coordination and R&D Center, Bursa 16245, Turkey

**Keywords:** bioplastics, poly(3-hydroxybutyrate), poly(lactic acid), soil, treatment sludge, vermicompost

## Abstract

In this study, the biodegradability of poly(lactic acid) (PLA), the most widely produced bioplastic, and poly(3-hydroxybutyrate) (PHB), known for its very biodegradability, was investigated in soil and soil amended with nitrogen sources, such as treatment sludge and vermicompost. Biodegradability was evaluated over 180 days by measuring the amount of carbon dioxide (CO_2_) and analyzing samples with scanning electron microscopy (SEM) and Fourier transform infrared spectroscopy (FTIR). PLA showed a low biodegradation (6%) in soil, but this increased to 40% in soil amended with treatment sludge and 45% in soil amended with vermicompost. PHB completely degraded within 90 days in soil; however, this process extended to 120 days in soil amended with vermicompost and 150 days in soil amended with treatment sludge. The organic and microbial content of the amendments enhanced PLA biodegradation, while PHB degradation slowed after 50 days as microorganisms prioritized other organic matter. SEM and FTIR analyses after 60 days showed more intense degradation of both bioplastics in soil amended with vermicompost. These findings highlight the potential of treatment sludge and vermicompost for improving bioplastic degradation, contributing to sustainable waste management and soil enhancement.

## 1. Introduction

With the growing population, the demand for plastic materials has increased, which has led to a rise in interest in sustainable materials. Due to the environmental risks posed by using traditional petroleum-based plastics, biodegradable bioplastics have emerged as a significant research area for sustainable development and environmental protection. However, long-term studies on this subject have not been adequately conducted [1,2,3].

Bioplastics are biodegradable polymers produced from renewable materials [4]. Poly(lactic acid) (PLA), which is among the polymerized biomonomers [5,6], is a semi-crystalline or amorphous, rigid thermoplastic and aliphatic polymer [7]. PLA is the most widely produced bioplastic and is considered an alternative to petroleum-based plastics [8,9,10]. PLA is commonly used in various fields, such as the packaging sector, medical applications [11], and food contact materials [12,13]. Another promising type of bioplastic based on renewable resources is polyhydroxyalkanoates (PHAs). PHA has significant potential, particularly in packaging, agriculture, textile, and biomedical applications, due to its biodegradability and biocompatibility [14]. Poly(3-hydroxybutyrate) (PHB) and its copolymer, polyhydroxybutyrate-co-hydroxyvalerate (PHBV), are the most significant polymers of the PHA family. There are various types of biodegradable plastics with differing degrees of biodegradability; among them, PHBs are the only materials that are 100% biodegradable [15].

The environment in which biodegradable plastics are placed or disposed of is a significant factor in their biological degradation [16,17]. Since biodegradable waste is commonly disposed of in soil environments, changes and effects in this area are being investigated. Additionally, aerobic composting environments are among the most widely studied degradation settings and serve as a standard waste-processing option [18]. Other notable environments include anaerobic sludge, freshwater, and seawater [19]. Soil environments generally contain a variety of microorganisms, such as *Chryseobacterium* sp., *Sphingobacterium* sp., *Stenotrophomonas pavanii*, *Pseudomonas geniculata*, and *Thermopolyspora flexuosa*, enabling better degradation of biodegradable plastics compared to environments such as water or air [16,20]. Biodegradation in soil occurs through the enzymatic breakdown of organic compounds that microorganisms can use as a carbon source [21]. However, environmental conditions such as soil temperature, moisture, and nutrients influence the reproduction of the soil microbiome and, consequently, affect degradation efficiency [22,23,24]. pH and oxygen levels are among the critical environmental factors to consider [25,26]. Soil amendments can improve the soil’s physical, chemical, and biological properties, enhancing nutrient availability and microbial activities in the soil [27]. Materials with soil-enhancing properties that increase organic matter content include manure, compost, treatment sludge, biosolids, and humic substances [28]. In cases where organic resources are insufficient, wastewater treatment sludge and compost with suitable properties are commonly used as alternative sources. Treatment sludge and compost benefit plant growth and soil fertility due to their content of essential nutrients such as nitrogen and phosphorus [29].

Many researchers have studied the biodegradation of biodegradable plastics under industrial composting conditions. For example, Karamanlioglu and Robson [30] found that PLA degraded by 53% in 57 days at 55 °C, Luo et al. [31] reported 78.9% degradation in 90 days at 58 °C, and Borelbach et al. [32] observed a reduction in fiber strength and molecular weight over four weeks at 58 °C. Additionally, many researchers, such as Weng et al. [33] and Seggiani et al. [34], have also studied the biodegradation of PHB in industrial composting environments. The biodegradation of biodegradable plastics is typically assessed under controlled laboratory conditions by measuring the conversion of organic carbon to carbon dioxide (CO_2_) in closed containers [35]. Under actual soil and composting conditions, it is not possible to measure CO_2_ emissions directly; therefore, techniques such as Fourier transform infrared spectroscopy (FTIR) and scanning electron microscopy (SEM) analysis are required to evaluate biodegradation. With standard laboratory tests, these measurements enable the evaluation of biodegradation in actual soil or compost conditions [36,37,38,39,40,41,42].

Investigating the degradation properties of PLA and PHB under different environmental conditions is crucial for both ecological sustainability and the real-world application of bioplastics. In this study, the degradation rates of these bioplastics were investigated in three different environments: soil, soil amended with treatment sludge, and soil amended with vermicompost. Previously, studies examined the biodegradation properties of PLA in soil [30,43], compost [44,45], and treatment sludge [46]; PHB in soil [47,48], compost [49,50], and treatment sludge [29,51,52,53]. However, studies specifically investigating bioplastics in environments such as compost-amended soil or treatment sludge-amended soil are quite limited. In a study, the biodegradability of PHBV in soil environment amended with 10% compost by weight at 25 °C was investigated [54]. In another study, the biodegradation of PLA in a soil–treatment sludge mixture (25:1) inoculated with fertilizer or wastewater sludge extract at 58 ± 2 °C was examined [55]. Additionally, a study investigated the biodegradation of PLA at 58 ± 2 °C in soils containing various microbial consortia, such as wastewater sludge from industries like dairy products, rice vermicelli, and coconut milk, as well as cow manure compost and green yard-waste compost [56]. Most research focused on degradation rates directly in compost or treatment sludge, while comprehensive studies on the degradation behavior in soils where these organic materials are applied are very limited. No studies examined the degradation behavior of PLA and PHB under controlled temperature conditions (28 °C) in soil environments amended with food industry treatment sludge or vermicompost nor compared the biodegradability of these bioplastics in such environments. This study aimed to investigate the innovative use of nitrogen-rich amendments like treatment sludge and vermicompost to significantly enhance PLA biodegradability and examined the degradation dynamics of PHB produced by a different method in amended soil environments. A commercial PLA in film form and PHB in multifilament yarn form, produced using a melt-spinning line method equipped with a specialized cooling apparatus, were used. The study will provide a novel framework for developing sustainable waste management strategies for bioplastics and other organic waste.

## 2. Materials and Methods

### 2.1. Materials

In this study, 500 mL capacity glass containers were used as reactors. The experiment aimed to simulate a real environmental scenario. The soil samples used in the study were obtained from a test field located on the campus of Bursa Uludag University (40°13′ 40,21870″ N–28°51′28,86311″ E). This soil is classified as clay loam in texture, containing 38.04% clay, 39.19% silt, and 22.77% sand. The hydrometer method was used for making texture analyses of soils [57]. The treatment sludge used in the study was sourced from a wastewater treatment plant associated with a food industry producing processed vegetables and fruits. It was employed as a soil amendment material. The compost used was a commercially available vermicompost derived from cow manure. The treatment sludge and vermicompost were dried, crushed, and sieved through a 2 mm mesh. Treatment sludge and vermicompost were applied to the soil samples at a rate providing 150 kg N/da of nitrogen. The general characteristics of the soil, treatment sludge, and vermicompost are presented in Table 1. The experiments were conducted in triplicates. It was determined that the total nitrogen (TN), ammonium nitrogen (NH_4_^+^-N), nitrate nitrogen (NO_3_^−^-N), and organic carbon contents of the treatment sludge and vermicompost were significantly higher compared to the soil.

To understand the effects of soil on biodegradation, cellulose (filter paper), commonly used as a positive reference in most international standards, was utilized [63,64]. The degradation of the positive reference material is used as an indicator of ongoing biological activity and decomposition. However, it is not a measure of biodegradation [65]. The PLA used in this study was obtained in pellet form from NatureWorks. A thermal press (POLMAK, PLM 30T, POLMAK Co., Ltd., Istanbul, Turkey) was used to produce films from the pellets. PLA films were prepared under 5 MPa pressure at 190 °C for 7 min. Before use, the films were cut into approximately 4 × 4 cm^2^ pieces (0.75 g) and stored at room temperature in the laboratory. PHB was provided by the Department of Textile Engineering at Bursa Uludag University [66]. The PHB materials were multifilament yarns consisting of 48 filaments. PHB yarns were cut into approximately 1 m pieces (0.25 g) and stored at room temperature. The carbon content of PLA and PHB are 50.6% and 57.2%, respectively. The elemental carbon analysis was performed using the Costech ECS 4010 Elemental Analyzer in an oxidation furnace at 1200–1050 °C for 25 min by TÜBİTAK Bursa Test and Analysis Laboratory, Bursa, Turkey. The properties of the biodegradable plastics used in this study are presented in Table 2.

### 2.2. Experimental Procedure

**Figure 1:**
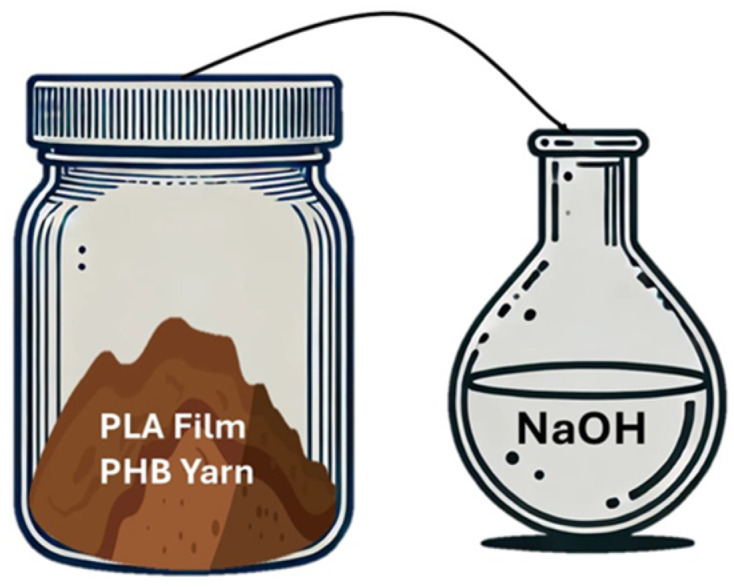
Experimental system of biodegradation of PLA films and PHB yarns.

The biodegradation studies were conducted in isolated containers within a ventilated incubator (JSR, JSGI-50T, JS Research Inc., Gongju-City, Republic of Korea) at 28 °C. PLA films and PHB-biodegradable multifilament yarns were incubated in soil, soil amended with treatment sludge, and soil amended with vermicompost for 180 days. In the experimental setup, 200 g of soil was placed at the base of glass reactors, and lime-stabilized treatment sludge (2.9 g) and vermicompost (4.2 g) were added to specific samples to provide nitrogen at a rate of 150 kg N/da. PLA films and PHB yarns were buried in the soil, soil with treatment sludge, and soil with vermicompost. Additionally, control samples were prepared to evaluate the behavior of soil, treatment sludge-amended soil, and vermicompost-amended soil during the incubation process without the influence of bioplastic samples. To ensure optimum conditions for microorganisms to degrade the samples, the ambient moisture was maintained at 70% of field capacity in accordance with the relevant standards. As shown in Figure 1, 100 mL of 0.2 N sodium hydroxide (NaOH) solution connected with the incubation reactors by polyethylene tubes was placed to capture CO_2_. The reactors were sealed and placed in a dark incubator [22,67]. On days 0, 2, 5, 8, 11, 15, 30, 45, 60, 75, 90, 120, 150, and 180, to determine the amount of CO_2_, carbon dioxide accumulated in the flask was taken with a solution of 50 mL and titrated with a solution of 0.2 M HCl with phenolphthalein and methyl orange indicator [68,69]. Moisture content was maintained constant by adding deionized water throughout the biodegradation test whenever the NaOH solution was titrated and replaced with a fresh one. These measurements were used to assess the biodegradability of the embedded samples. The experiments were conducted in triplicate for each condition, and the experimental setup is summarized in Table 3.

### 2.3. Characterization

In this study, the methods used to determine the biodegradation process of bioplastics include CO_2_ emission measurement and the examination of their surface morphology and chemical structures. These methods play a significant role in understanding how long plastics take to decompose in the environment. To assess the mineralization of the samples, residual samples were carefully extracted from the soil, washed with ethanol and distilled water, and dried at 28 °C for 4 h. The morphological and chemical properties of the dried samples were analyzed using SEM and FTIR at the beginning and end of the incubation period.

#### 2.3.1. Calculation of CO_2_ Emissions

The gas emission measurement test was carried out in accordance with the ASTM D5988-18 standard [70]. The key parameter indicating biodegradation in the experiment is the amount of CO_2_ released in a closed system. For this purpose, a NaOH solution, which acts as an adsorbent for CO_2_, was added to each reactor. These solutions were subsequently titrated with HCl solution. The following equation was used to calculate the theoretical CO_2_ (CO_2_(t)) (g) in the total dry plastic material:

(1) $${CO}_{2}(t)=M_{t}\cdot C_{t}\cdot \frac{44}{12}$$

*M_t_* represents the plastic material added to the soil medium (g). The samples’ total organic carbon (*C_t_*) was determined through elemental analysis. The biodegradation rate for each test material was calculated as a percentage of the total theoretical CO_2_.

(2) $$Biodegradation\;\left(\%\right)=\frac{g^{{CO}_{2}}-g^{{CO}_{2}}b}{{CO}_{2}(t)}\cdot 100$$

$g^{{CO}_{2}}$ and $g^{{CO}_{2}}b$ represent the CO_2_ released from the sample (g) and from the control samples (g), respectively [71].

#### 2.3.2. Fourier Transform Infrared Spectroscopy (FTIR) Analysis

FTIR is one of the most reliable and cost-effective methods for identifying the main components of a plastic material. One of FTIR’s strongest features is its ability to identify degradation–oxidation within plastics. Since PHB yarns underwent high mineralization levels (>90%) within 75 days, FTIR analysis was conducted on the 60th day. For PLA films, as their structural integrity was preserved mainly, the analysis was performed on the 180th day. FTIR spectra were obtained using a PerkinElmer Spectrum 100 device, (Waltham, MA, USA) with an ATR module to examine the functional groups in the structures of PHB yarns and PLA films. The analysis was conducted in the wavelength range of 400–4000 cm^−1^ with a resolution of 0.5 cm^−1^. The evaluation of the spectra was performed using OriginPro software (OriginPro, Version 2024b. OriginLab Corporation, Northampton, MA, USA).

#### 2.3.3. Scanning Electron Microscopy (SEM)

Morphological degradation was observed using an SEM device (ZEISS EVO 40 Carl Zeiss NTS GmbH, Oberkochen, Germany). The fracture surfaces of PHB yarns on the 60th day and PLA films on the 180th day were examined using SEM at an acceleration voltage of 20 kV. Images were obtained at 2000× and 1000× magnification for PLA and PHB, respectively.

## 3. Results and Discussion

### 3.1. Evaluation of Biodegradation Through CO₂ Measurement

Assessing biodegradation through CO_2_ measurement is crucial for understanding the environmental impacts of biodegradable materials and the efficiency of biodegradation processes. In this study, the biodegradation of cellulose, used as a reference material, exceeded 90% after two months. The high degradation rate of cellulose, used as a reference material, indicated that the soil with the chemical properties listed in Table 1 was suitable and had a high level of microbial activity. The degradation process of PLA and PHB was monitored over a 180-day incubation period in this study.

**Figure 2:**
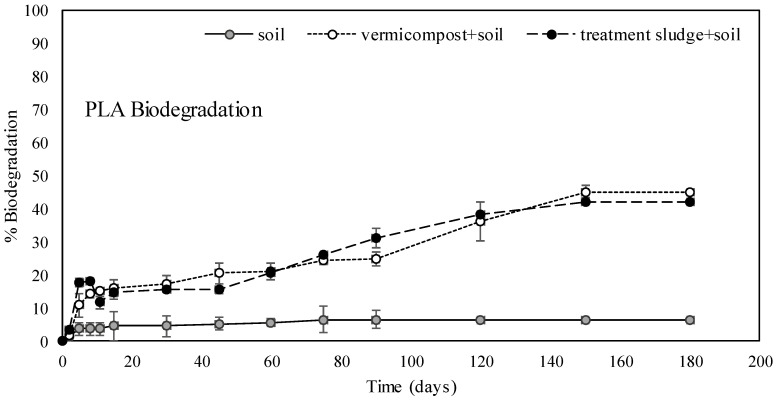
Biodegradation rates of PLA in soil, treatment sludge-amended soil, and vermicompost-amended soil environments.

Although PLA is a biodegradable polymer, significant differences in its biodegradation rate are observed under various environmental conditions. Figure 2 shows the biodegradation rates of PLA in soil, soil amended with treatment sludge, and soil amended with vermicompost. In the soil environment, the biodegradation rate of PLA remained very low (approximately 6%) even after 180 days. In contrast, the biodegradation rate increased nearly sevenfold, reaching about 40%, in the soil amended with treatment sludge. The highest biodegradation was observed in the soil amended with vermicompost, where approximately 45% of PLA was biologically degraded after 180 days. These results demonstrated that the biodegradation potential of PLA varied significantly depending on the environmental conditions. It was specifically determined that environments rich in organic content, nitrogen, and microorganisms, such as treatment sludge and vermicompost, significantly accelerated the biodegradation process. In this study, the organic materials added to the soil were determined to have substantially higher nitrogen contents than the soil. While the total nitrogen content of the soil was 0.165%, it was determined to be 3.08% for treatment sludge and 2.87% for vermicompost (Table 1). Similarly, in a study conducted by Pattanasuttichonlakul et al. [56], different nitrogen sources were applied to the soil at an 80/20 ratio to investigate the biodegradability of commercial PLA drinking cups. The researchers concluded that nitrogen sources accelerated biodegradation. They found that PLA was degraded entirely after being buried under aerobic thermophilic conditions for 15 days in soil amended with wastewater sludge from a dairy factory, which had a high total nitrogen content (4.39%). Likewise, they observed complete degradation within 30 days in soil amended with wastewater sludge from a coconut milk factory, which also had a similarly high nitrogen content (3.99%). These results showed higher degradation rates compared to this study, likely due to the incubation at thermophilic temperatures and the use of weight loss to determine biodegradability. Although determining biodegradability through weight loss provides an indication, it does not yield definitive results [72].

In their study, Palsikowski et al. [64] reported a biodegradation rate of 16% for PLA in soil after 180 days. Similarly, Satti et al. [73] observed approximately 10% biodegradation in the soil at 30 °C after 150 days, while Muniyasamy et al. [74] found a biodegradation rate of about 4% in soil after 200 days. These values are comparable to the biodegradation rate of PLA (6%) determined in this study. Rudnik and Briassoulis [75] reported that, regardless of their form, PLA materials required more than 11 months to biodegrade in soil under Mediterranean climate conditions.

Many researchers investigated the biodegradation of PLA in compost environments. However, most of these studies were conducted under thermophilic conditions in industrial composting settings. For PLA in 100% compost environments, Tabasi and Ajji [76] reported approximately 70% degradation at 55 °C within 28 days, while Stloukal et al. [45] measured 70.86% degradation in 90 days at 58 °C. Kale et al. [71] observed 84% degradation at 65 °C within 58 days, and Mihai et al. [77] reported 60% degradation at 58 °C within 30 days. In this study, the degradation of PLA in vermicompost-amended soil at 28 °C was approximately 45%. The primary reason for the lower degradation rate compared to literature studies was that other studies were conducted at thermophilic temperatures, whereas this study was conducted at ambient temperature. Furthermore, in other studies, the biodegradability of PLA was determined only in compost environments, whereas in this study, biodegradability was determined in a compost-amended soil environment. According to Karamanlioglu et al. [17], high temperatures (50–60 °C) and moisture in compost environments promote the rapid degradation of PLA, and the high microbial density in compost accelerates its breakdown further. Similarly, treatment sludge environments also support the rapid degradation of PLA. The microbial richness and suitable temperature and moisture conditions in treatment sludge significantly enhance PLA biodegradation. Ren et al. [78] reported that PLA completely degraded within one month in a 100% anaerobic digestion sludge system.

**Figure 3:**
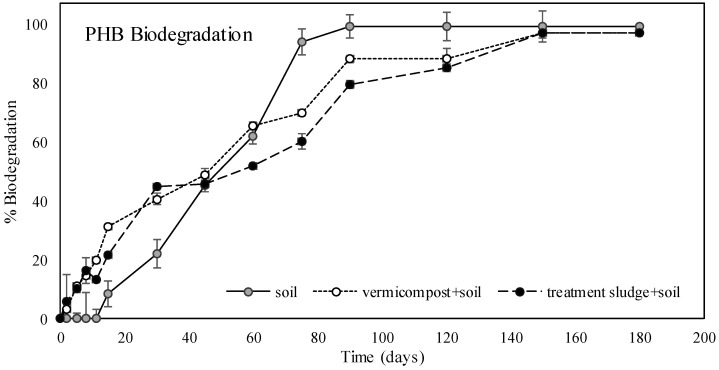
Biodegradation rates of PHB in soil, treatment sludge-amended soil, and vermicompost-amended soil environments.

Figure 3 illustrates the biodegradation rates of PHB over 180 days in three different environments: soil, soil amended with treatment sludge, and soil amended with vermicompost. The biodegradation of PHB in soil started relatively slow but accelerated after the 15th day, exceeding 90% by the 75th day and reaching complete degradation by the 90th day. In vermicompost-amended soil, biodegradation began much faster; by the 30th day, approximately 20% biodegradation was observed in the soil, whereas 40% biodegradation was recorded in vermicompost-amended soil, with near-complete degradation achieved by the 120th day. Similarly, in treatment sludge-amended soil, biodegradation began rapidly, with approximately 45% biodegradation observed by the 30th day, reaching around 90% after the 120th day and near-complete degradation by the 150th day. Overall, adding treatment sludge and vermicompost to soil enhanced microbial activity and enzyme activity, significantly accelerating PHB biodegradation during the first 45 days while these organic materials remained abundant. However, PHB degradation occurred more quickly in plain soil (90 days). The faster biodegradation of PHB in plain soil compared to soil + treatment sludge and soil + vermicompost can be attributed to its simpler and more uniform environment, with lower organic content and fewer competing substrates. Palucha et al. [79] stated that PHB served as the primary substrate for soil microorganisms. In contrast, the enriched and heterogeneous conditions in the latter environments may lead to nutrient prioritization, potential anaerobic zones, and fluctuating microbial activity, resulting in slower degradation. The biodegradation rate in treatment sludge and vermicompost being lower than in soil after approximately the first 50 days could have been that the microorganisms in these organic materials prioritized other organic substances over PHB. In other words, PHB might not be microorganisms’ preferred primary energy source, leading to a slower degradation process. The study by Tokiwa and Calabia [80] demonstrated that the degradation of PHB by microorganisms slowed down when other organic carbon sources were available in the environment. Microorganisms first target more easily degradable organic materials, particularly in organic matter-rich environments such as compost or treatment sludge. Also, Figure 3 shows steps that appear on the biodegradation kinetic curves of PHB. The steps observed on the biodegradation kinetic curves of PHB in the soil + treatment sludge and soil + vermicompost environments could result from factors specific to these environments. Rich microbial diversity and organic content in these substrates may lead to fluctuations in microbial activity, changes in nutrient availability, localized anaerobic conditions, and phased PHB degradation (e.g., surface erosion followed by fragmentation). Additionally, their heterogeneity creates microenvironments with varying degradation rates. In contrast, plain soil, with lower microbial diversity and organic matter, provides more uniform conditions, resulting in stable and linear biodegradation. Further studies on microbial dynamics and nutrient profiles could clarify these patterns.

There are various studies on the biodegradability of PHB in soil. Rehman et al. [81] reported a 57.3% biodegradation rate for PHB polymers in soil after 56 days. Similarly, in this study, PHB was found to biodegrade by 58.6% in soil by day 60. Pérez-Arauz et al. [82] determined an 82% degradation rate within 80 days. In this study, it was determined that PHB biodegraded by 90% in soil on the 75th day and completely degraded on the 90th day, and these results were found to be comparable with the literature. However, Rudnik and Briassoulis [63] reported approximately 90% degradation of PHB films in soil after one month. Altaee et al. [48] observed 100% degradation of PHB nanofibers within three weeks, while Kim et al. [83] reported complete degradation of PHB films in soil within two weeks. However, the PHB filaments in this study generally exhibit a medium-to-high crystalline structure (~55%). The crystalline structure hinders biodegradation because microorganisms find it more challenging to break down crystalline regions than amorphous ones [84]. Gasparyan et al. [85] examined the impact of PHB’s crystalline structure on degradation processes and mechanical properties, noting that an increase in crystalline regions in PHB films enhanced resistance to biological degradation and slowed down the degradation rate. Furthermore, it was pointed out that the abundance of crystalline structure also improved the mechanical strength of the film, giving it properties like synthetic polymers. It was also highlighted that the production method significantly influenced the surface morphology and structure of the material. In this study, PHB melted at high temperatures while the melt-spinning process was rapidly cooled, leading to a more orderly crystalline structure. This enhanced the mechanical strength of the filaments but also reduced the biodegradation rate. Armentano et al. [86] indicated that the mechanical durability of PHB could be improved through various processing methods, with the melt-spinning method being particularly effective for fiber production.

Several studies investigated the biodegradability of PHBs in different environments. Weng et al. [33] reported a 79.7% biodegradation rate for PHB in 110 days under thermophilic conditions in mature compost obtained from a municipal organic waste composting facility. Similarly, Seggiani et al. [34] determined 92% degradation of PHB in 78 days under thermophilic conditions (58 °C) in compost derived from urban solid waste. In this study, a degradation rate of 88% was observed in compost-amended soil over 90 days despite the soil not being entirely composed of compost. These results were consistent with those reported in the literature. Gutierrez-Wing et al. [29] examined the anaerobic biodegradation of PHB films in municipal sewage sludge and found a biodegradation rate exceeding 95% within 8 weeks. Huda et al. [87] investigated the anaerobic degradation of PHB pellets in activated sludge under different temperatures during sludge digestion. They observed a degradation rate of 20% at 20 °C in 7 days, 30% at 30 °C in 4 days, and 78% at 37 °C in 3 days. According to Al-Khattaf et al. [53], PHB films degraded by 96% in 8 days at 30 °C in a sequencing batch biofilm reactor. In this study, PHB degraded approximately 10% in 5 days and completely degraded in 150 days under aerobic conditions in soil amended with treatment sludge at 28 °C. This lower degradation rate compared to the literature can be attributed to the filament structure and medium-to-high crystalline nature of the PHB used, aerobic degradation at ambient temperature, and the fact that the environment was not entirely composed of treatment sludge. No studies have investigated PHB biodegradability in soil amended with treatment sludge applied at a rate that provides 150 kg N/da of nitrogen.

### 3.2. Morphological Evaluation of Biodegradation

The surface morphology of polymers during degradation in soil environments was examined using SEM. Since biodegradation in soil was high (over 90%) after 75 days, PHB yarns were analyzed after 60 days of incubation. In contrast, PLA samples were subjected to morphological evaluation at the end of 180 days.

**Figure 4:**
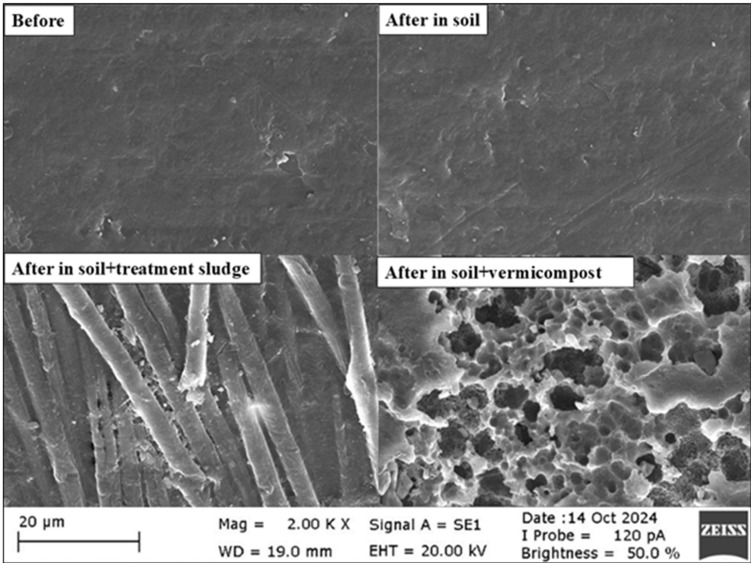
SEM micrographs for PLAs before and after degradations in different environments.

The SEM images of PLA samples before and after 180 days of incubation in soil, treatment sludge-amended soil, and vermicompost-amended soil are shown in Figure 4. As indicated by the SEM analysis results, while the surface of PLA was smooth and homogeneous before incubation, minor changes were observed after 180 days of degradation in the soil. The PLA surface exhibited the beginning of roughness and fine lines. The PLA surface showed significant degradation in the treatment sludge-amended soil, forming longitudinal lines and cracks, indicating polymer disintegration. This suggests that the organic matter and microorganisms introduced with the treatment sludge accelerated the degradation process. The most intense degradation of PLA was observed in vermicompost-amended soil, as revealed by the SEM images. The PLA surface became entirely irregular, with extensive porosity and fragmentation. It was evident that the compost environment caused intense degradation of PLA, and the microbial activity induced significant alteration in the surface morphology. These morphological findings demonstrated that adding nutrient-rich soil amendments, such as treatment sludge and compost, enhanced PLA biodegradation. Walczak et al. [88] evaluated the biodegradability of PLA in different environments. They observed that after 14 days of incubation at 20 °C, PLA degraded more effectively in compost environments than in soil. Muniyasamy et al. [74] also reported similar porous structures in PLA under thermophilic composting conditions. In their study, Lee et al. [89] investigated the morphological changes in PLA under anaerobic digester sludge conditions. After 30 days of incubation, they observed physical degradation in the form of holes and pits on the PLA surface. This type of morphology is attributed to the hydrolytic degradation of PLA molecules in the soil environment. Moisture from the soil penetrates the PLA matrix and hydrolyzes the ester groups in the PLA main chain [90]. Many researchers have noted that the degradation mechanism of PLA was generally characterized by initial chemical hydrolysis followed by microbial activity [30,91,92]. Ren et al. [78] studied the biological degradation of PLA in digested sludge and found that after 39 days, cracks and pits had formed on the PLA surface due to microbial activity. These prominent holes and irregular degradations on the PLA surface indicate that microbial activity had broken down the PLA polymer chains, compromising its structural integrity. In another study under thermophilic conditions, where equal amounts of soil and sludge were mixed, a biodegradation test of PLA revealed numerous pores, irregular roughness, and cracks on the PLA surface after 90 days [93].

In this study, no significant surface corrosion was detected on PLA in soil after 180 days at ambient temperature. Similarly, Weng et al. [94] reported that no considerable surface corrosion was observed on PLA films in soil after 4 months. These results indicate that adding nutrient-rich additives enhances the biodegradation of PLA. It was clearly understood that the degradation process was accelerated in soils treated with treatment sludge and compost. These findings demonstrated that the degradation profile of PLA varied significantly depending on environmental conditions. After 180 days of incubation, SEM images were consistent with the calculated biodegradability results.

**Figure 5:**
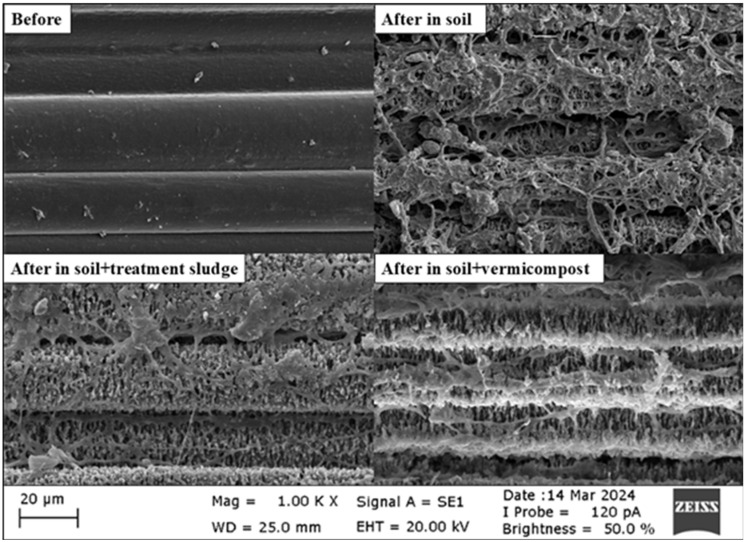
SEM micrographs for PHBs before and after degradation in different environments.

The SEM photographs showing the morphological changes during the degradation process of PHB films are presented in Figure 5. Before degradation, PHB surfaces were generally observed to be smooth and uniform. No significant cracks or holes were detected on the surface, and the material’s internal structure was homogeneous. The polymer chains were arranged in an orderly manner. After a 60-day incubation period, the surfaces of PHB filaments in all environments were observed to have become eroded and rough. The filaments’ pores, cavities, grooves, protrusions, and tears indicated an irregular surface structure.

Significant changes in the surface structure of PHB were observed after degradation in soil. During the degradation process, the polymer was degraded by microorganisms, resulting in irregular cavities, pores, and fibrous, network-like structures on the surface. The protrusions and the fusion of yarns in the images indicate the colonization of microorganisms and enzymatic activity in the soil environment. Fungal hyphae and other microorganisms’ filamentous structures have become prominent on the PHB surface. In a study by Lopez-Llorca et al. [95], the biological degradability of PHA films in soil was tracked using SEM after 45 days. SEM images clearly showed fungal hyphae and smaller filaments (likely actinomycetes) colonizing the surface of the PHA films. According to Feijoo et al. [96], fungi hyphae appeared in PHBV at 15 days. Additionally, the fragmentation and layered structure resulting from the breaking of polymer chains were noticeable. These structural changes indicated that PHA was degrading under biological and chemical processes, transforming from an initially smooth surface into a more complex one. Similarly, according to Altaee et al. [48], SEM images of PHB nanofibers in soil revealed an irregular surface with pores, cavities, grooves, elongated structures resembling fungal hyphae, spherical structures resembling bacteria, and visible ruptures of many nanofibers.

In soil amended with treatment sludge, Figure 5 shows that as PHB degrades due to biological or environmental effects, wear, holes, and microcracks form on the surface. However, the structure in the treatment sludge-amended soil was relatively smoother, arranged in horizontal lines. Degradation was limited to fewer irregular and rough areas. While there was some roughness and fragmentation on the surface, the overall structure was maintained. Compared to PHB yarns incubated in treatment sludge-amended soil, PHB yarns incubated in soil became more eroded after two months of biodegradation. This could have been due to the high nutrient content in the treatment sludge, which increased competition among microorganisms, causing them to delay degrading PHB by first utilizing the available nutrients in the treatment sludge. Similarly, in a study by Gómez and Michel [97], the biodegradability of co-polyester + corn-based plastics was investigated in both soil (at 20 °C) and anaerobic digesters processing urban sewage sludge at 37 °C in a methanogenic active sludge. They found that 20% of the co-polyester + corn-based plastics degraded in the soil after 20 days of incubation, whereas it took 50 days to reach the same level in sludge from the anaerobic digesters. In this study, the calculated biodegradability in soil after 60 days was approximately 62%, while in soil amended with treatment sludge, it was 52%. SEM images also confirm this result.

Upon examining the SEM images in soil amended with vermicompost, the horizontally arranged structure remains prominent, but it is observed that the upper layers are eroded and thinned (Figure 5). Corrêa et al. [98] exposed injected PHB samples to biodegradation in a soil environment simulated with cow manure for 3 months. According to the results of the study, biodegradation occurred in a layer-by-layer manner. After 3 months, the top layer of PHB completely degraded, and degradation began in a new layer. Compost contains various organisms, cellulose, and other enzymes. Enzymatic hydrolysis initially destroys the outer layers of the filament. Then, organisms convert the inner part of the fibers into hydrolysates [99]. Similarly, in the images of soil amended with vermicompost in Figure 5, significant erosion and dissolution of the material were observed on the upper part of the fibers. This suggested that microorganisms were attacking the fiber surface and breaking the fibers during biodegradation. Here, the degradation was slower and more gradual due to the effect of the compost. In a study by Weng et al. [33], the biological degradation behavior of five different PHA films was examined. In the SEM images of the PHA films, numerous pores and degradation were detected under compost conditions after 30 days. Among all the PHAs, they found that PHB had the most regular structure and the highest crystallinity. In this study, the calculated biodegradability in soil after 60 days was approximately 62%, while in soil amended with vermicompost, it was about 66%. Compost might have enhanced degradation due to the fungi and actinomycetes it contained. The SEM images were consistent with the 60-day biodegradation results.

### 3.3. Evaluation of Biodegradability Using Fourier Transform Infrared Spectroscopy (FTIR)

**Figure 6:**
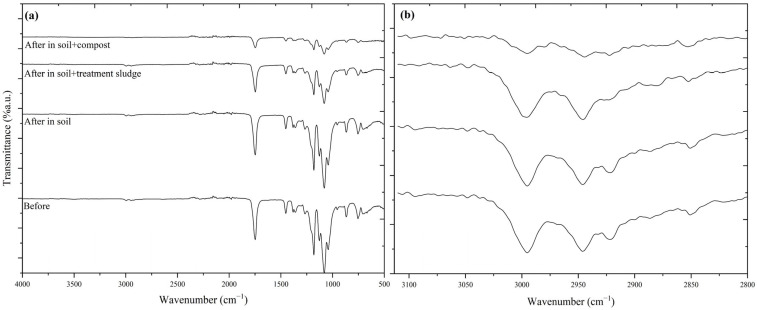
FTIR for PLAs before and after degradation in different environments: (**a**) wavenumber 4000–500 cm^−1^ and (**b**) wavenumber 3113–2800 cm^−1^.

The FTIR spectra of PLA before and after 180 days of degradation are shown in Figure 6. In the PLA spectrum, a strong peak at 1748 cm^−1^ corresponded to the carbonyl group (C=O) stretching in polyester [100,101]. Additionally, characteristic stretching signals of the –CH group were observed at 2995 cm^−1^ and 2946 cm^−1^ (Figure 6b) [102,103]. The band attributed to the deformation of the CH₃ group was found at 1453 cm^−1^, while the band corresponding to the symmetric CO stretching was located at 1180 cm^−1^. Furthermore, three bands associated with symmetric C-O-C stretching appeared at 1127, 1080, and 1042 cm^−1^. These characteristic bands of PLA were consistent with the literature [104,105].

The carbonyl peak around 1750 cm^−1^ showed the greatest reduction in the vermicompost-amended soil environment. Treatment sludge also accelerated the degradation process, resulting in structural changes in PLA. After 180 days, in the soil amended with vermicompost, the ester peaks in the 1180–1260 cm^−1^ range had significantly weakened or almost disappeared. This indicates that PLA underwent more rapid and advanced biodegradation in the compost environment compared to other conditions. Additionally, there was a notable reduction in the C–H stretching vibrations in the 2900–3000 cm^−1^ range, indicating a significant breakdown of methyl and methylene groups. The FTIR results revealed that PLA degradation was at its highest levels with the addition of vermicompost, followed by treatment sludge addition, which also showed relatively high degradation. In contrast, degradation in the soil environment alone was minimal. The FTIR findings aligned with the calculated biodegradation results.

**Figure 7:**
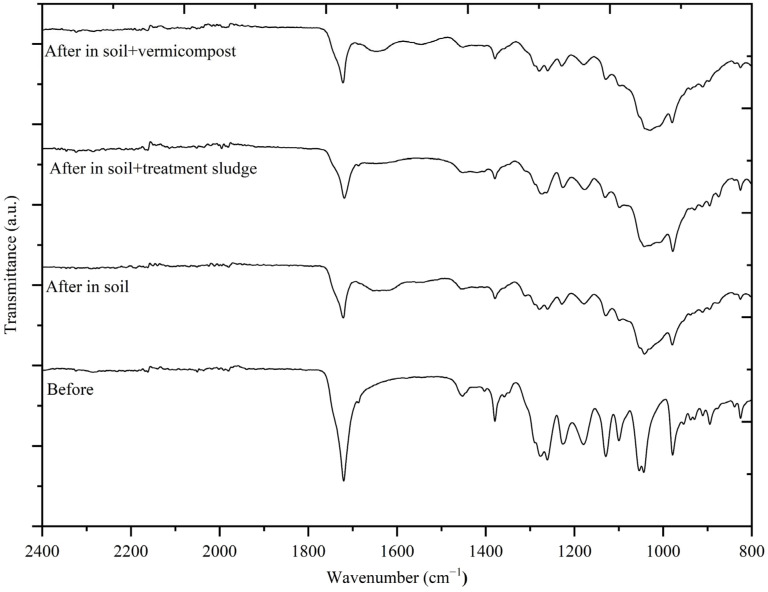
FTIR for PHBs before and after degradation in different environments.

The FTIR spectra of PHBs before and after 60 days of degradation are presented in Figure 7. Various characteristic spectral features were observed in the spectra of all samples. A notable feature is the sharp absorption band around 1720 cm^−1^, associated with the symmetric C=O stretching in aliphatic esters. The mineralization of PHB primarily occurs due to enzymatic and chemical hydrolysis, resulting in the cleavage of ester bonds [97]. Before biodegradation, the band at 1720 cm^−1^ shifted after 60 days of biodegradation to 1721 cm^−1^ in soil, 1718 cm^−1^ in soil amended with treatment sludge, and 1721 cm^−1^ in soil amended with vermicompost. This shift indicates the breakdown of ester groups in the polymer due to biodegradation, signifying the gradual degradation of the polymer. Weng et al. [33] similarly investigated the biodegradability of PHB under controlled composting conditions over 30 days and reported a comparable result for this band (1725–1724 cm^−1^).

The bands around 1130 and 1276 cm^−1^ are associated with C–O–C groups [106]. Before degradation, these peaks exhibit a more pronounced and sharp structure. After degradation, the amplitude and prominence of these bands have generally decreased. Among the samples, the most significant reduction in these bands due to degradation was observed in soil amended with vermicompost, followed by untreated soil, and then soil amended with treatment sludge. This indicated that PHB underwent the highest level of biodegradation in vermicompost-amended soil, with significant cleavage of ester bonds. These findings are aligned with the calculated biodegradation rates.

The signals at 1455 cm^−1^ and 1382 cm^−1^ are associated with the asymmetric stretching peak and the symmetric deformation peak of methyl (–CH3) and methylene (–CH2) groups, respectively [107]. In PHB yarns, these signals (observed at 1453 and 1379 cm^−1^) were initially strong but decreased in intensity as degradation progressed. The most significant reduction in the intensity of these signals was observed in vermicompost-amended soil, providing strong evidence that compost accelerated degradation. In contrast, the crystalline structure of the signals in treatment sludge-amended soil was more pronounced compared to other environments, indicating that biodegradation proceeds more slowly in these conditions.

According to Weng et al. [33] and Luo and Netravali [108], FTIR results showed that the biodegradation of PHA and PHBV under compost conditions primarily occurred due to microbial erosion, which initiated at the surface layer and gradually penetrated the interior of the plastic. Similarly, in this study, vermicompost-amended soil exhibited comparable results, whereas the structure of PHB in treatment sludge-amended soil remained relatively intact.

## 4. Conclusions

In this study, the biodegradation processes of PLA and PHB in different soil environments were evaluated, and the effects of adding organic additives such as treatment sludge and vermicompost to the soil were investigated. The findings showed that these additives significantly increased the biodegradation rate, particularly for PLA, by enhancing the soil’s microbial activity, organic matter, and nitrogen content. According to the results, at 28 °C over 180 days, the biodegradation rate of PLA in plain soil remained relatively low (6%), whereas this rate increased approximately sevenfold in soils amended with treatment sludge and vermicompost, reaching 40% and 45%, respectively. The biodegradation process of PHB was completed within 90 days in plain soil. However, in soils amended with treatment sludge and vermicompost, the addition of these organic materials accelerated biodegradation during the first 50 days, although the total time required for complete degradation of the polymer was longer. SEM and FTIR analyses confirmed that the biodegradation processes caused significant changes in the polymers’ surface morphology and chemical structures. Vermicompost application, in particular, created a more substantial degradation effect on the chemical structures of both PLA (after 180 days) and PHB (after 60 days). In conclusion, this study provides important insights for improving the environmental compatibility of bioplastics, developing sustainable waste management strategies, and evaluating the potential use of treatment sludge and vermicompost in soil improvement applications. It may also be possible to increase the biodegradation of bioplastics under different environmental conditions by adding various organic additives to the soil and adjusting their proportions.

## Figures and Tables

**Table 1:** Characterization of soil, treatment sludge, and vermicompost.

Parameters	Soil	Treatment Sludge	Vermicompost	Method
pH, 25 °C (1:5)	8.38 ± 0.03	7.11 ± 0.02	6.92 ± 0.03	Determined using a Mettler-Toledo pH meter in a 1:5 distilled water extract [58]
EC, µS/cm 25 °C (1:5)	144 ± 3.32	5650 ± 4.55	7626 ± 6.56	Determined using a Mettler-Toledo EC meter in a 1:5 distilled water extract [59]
Total N, %	0.165 ± 0.01	3.08 ± 0.19	2.87 ± 0.18	Samples were combusted using the Kjeldahl method, and the steam distillation technique was applied [60]
NH_4_^+^-N, mg kg^−1^	46.3 ± 2.52	114.2 ± 5.09	642.4 ± 6.09	Samples extracted with 2 Mol KCl were analyzed using steam distillation with MgO [61]
NO_3_^−^-N, mg kg^−1^	89.0 ± 3.57	178.4 ± 0.01	619.2 ± 4.62	Samples extracted with 2 Mol KCl were analyzed using steam distillation with MgO and Devarda’s alloy [61]
Organic C (%)	6.83 ± 0.27	8.56 ± 0.12	15.75 ± 0.60	After oxidation with a potassium dichromate solution, determined spectrophotometrically at 590 nm [62]

**Table 2:** Properties of the bioplastic samples.

Samples	Production/Preparation	Origin
PLA	Product code 2003D, has a molecular weight of 35 × 10^4^ g/mol, a glass transition temperature (Tg) of 61 °C, and a melting temperature (Tm) of 169 °C. Carbon (C): 50.6%. The film was prepared using a thermal press at 190 °C under 5 MPa pressure for 7 min.	NatureWorks
PHB	PHB multifilament yarn was produced using the melt spinning line method with a specialized cooling apparatus. (Winding speed [m/min]: 500, Draw Ratio: 1, Cooling apparatus was utilized). Molecular weight: 600 kDa, Crystallization Degree (X_a_): 55%, Viscosity (dL/g): 2.70, Melting temperature (°C): 176, Melt flow index (180 °C—2.16 kg) [g/10 min]: 10.74 [66]. Carbon (C): 57.2%.	Bursa Uludag University, Department of Textile Engineering

**Table 3:** Experimental setup.

Number of Reactors	Content	Scope
3	Soil	Determining the background activity of the soil (control reactor)
3	Soil + treatment sludge	Determining the background activity of soil and treatment sludge (control reactor)
3	Soil + vermicompost	Determining the background activity of soil and vermicompost (control reactor)
3	Soil and PLA	Observing the biodegradability of PLA in soil
3	Soil and PHB	Observing the biodegradability of PHB in soil
3	Soil + treatment sludge and PLA	Comparing the biodegradability of PLA using soil amendments
3	Soil + treatment sludge and PHB	Comparing the biodegradability of PHB using soil amendments
3	Soil + vermicompost and PLA	Comparing the biodegradability of PLA using soil amendments
3	Soil + vermicompost and PHB	Comparing the biodegradability of PHB using soil amendments

## Data Availability

The authors confirm that the data supporting the findings of this study are either available within the article or can be obtained upon request.

## References

[1] Brodhagen M., Goldberger J.R., Hayes D.G., Inglis D.A., Marsh T.L., Miles C. (2017). Policy Considerations for Limiting Unintended Residual Plastic in Agricultural Soils. Environ. Sci. Policy.

[2] Steinmetz Z., Wollmann C., Schaefer M., Buchmann C., David J., Tröger J., Muñoz K., Frör O., Schaumann G.E. (2016). Plastic Mulching in Agriculture. Trading Short-Term Agronomic Benefits for Long-Term Soil Degradation?. Sci. Total Environ..

[3] Rydz J., Musioł M., Zawidlak-Węgrzyńska B., Sikorska W., Grumezescu A.M., Holban A.M. (2018). Chapter 14—Present and Future of Biodegradable Polymers for Food Packaging Applications. Handbook of Food Bioengineering.

[4] Cucina M., De Nisi P., Trombino L., Tambone F., Adani F. (2021). Degradation of Bioplastics in Organic Waste by Mesophilic Anaerobic Digestion, Composting and Soil Incubation. Waste Manag..

[5] Rudin A., Choi P., Rudin A., Choi P. (2013). Chapter 13—Biopolymers. The Elements of Polymer Science & Engineering.

[6] Babu R.P., O’Connor K., Seeram R. (2013). Current Progress on Bio-Based Polymers and Their Future Trends. Prog. Biomater..

[7] Henton D., Gruber P., Lunt J., Randall J., Mohanty A.K., Manjusri Misra L.T.D. (2005). Polylactic Acid Technology. Natural Fibers, Biopolymers, and Biocomposites.

[8] Yusoff N.H., Pal K., Narayanan T., de Souza F.G. (2021). Recent Trends on Bioplastics Synthesis and Characterizations: Polylactic Acid (PLA) Incorporated with Tapioca Starch for Packaging Applications. J. Mol. Struct..

[9] Ülger-Vatansever B., Onay T.T., Demirel B. (2024). Evaluation of Bioplastics Biodegradation Under Simulated Landfill Conditions. Environ. Sci. Pollut. Res. Int..

[10] Kirchkeszner C., Petrovics N., Tábi T., Magyar N., Kovács J., Szabó B.S., Nyiri Z., Eke Z. (2022). Swelling as a Promoter of Migration of Plastic Additives in the Interaction of Fatty Food Simulants with Polylactic Acid- and Polypropylene-Based Plastics. Food Control.

[11] Kalita N.K., Bhasney S.M., Mudenur C., Kalamdhad A., Katiyar V. (2020). End-of-Life Evaluation and Biodegradation of Poly(Lactic Acid) (PLA)/Polycaprolactone (PCL)/Microcrystalline Cellulose (MCC) Polyblends Under Composting Conditions. Chemosphere.

[12] Petrovics N., Kirchkeszner C., Tábi T., Magyar N., Kovácsné Székely I., Szabó B.S., Nyiri Z., Eke Z. (2022). Effect of Temperature and Plasticizer Content of Polypropylene and Polylactic Acid on Migration Kinetics into Isooctane and 95 *v*/*v*% Ethanol as Alternative Fatty Food Simulants. Food Packag. Shelf Life.

[13] Petrovics N., Kirchkeszner C., Patkó A., Tábi T., Magyar N., Kovácsné Székely I., Szabó B.S., Nyiri Z., Eke Z. (2023). Effect of Crystallinity on the Migration of Plastic Additives from Polylactic Acid-Based Food Contact Plastics. Food Packag. Shelf Life.

[14] Sudesh K., Abe H., Doi Y. (2000). Synthesis, Structure and Properties of Polyhydroxyalkanoates: Biological Polyesters. Prog. Polym. Sci..

[15] Getachew A., Woldesenbet F. (2016). Production of Biodegradable Plastic by Polyhydroxybutyrate (PHB) Accumulating Bacteria Using Low Cost Agricultural Waste Material. BMC Res. Notes.

[16] Emadian S.M., Onay T.T., Demirel B. (2017). Biodegradation of Bioplastics in Natural Environments. Waste Manag..

[17] Karamanlioglu M., Preziosi R., Robson G.D. (2017). Abiotic and Biotic Environmental Degradation of the Bioplastic Polymer Poly(Lactic Acid): A Review. Polym. Degrad. Stab..

[18] Ruggero F., Gori R., Lubello C. (2019). Methodologies to Assess Biodegradation of Bioplastics during Aerobic Composting and Anaerobic Digestion: A Review. Waste Manag. Res. J. Int. Solid Wastes Public Clean. Assoc. ISWA.

[19] Shruti V.C., Kutralam-Muniasamy G. (2019). Bioplastics: Missing Link in the Era of Microplastics. Sci. Total Environ..

[20] Mistry A.N., Kachenchart B., Wongthanaroj A., Somwangthanaroj A., Luepromchai E. (2022). Rapid Biodegradation of High Molecular Weight Semi-Crystalline Polylactic Acid at Ambient Temperature via Enzymatic and Alkaline Hydrolysis by a Defined Bacterial Consortium. Polym. Degrad. Stab..

[21] Lee S.Y. (1996). Bacterial Polyhydroxyalkanoates. Biotechnol. Bioeng..

[22] Pischedda A., Tosin M., Degli-Innocenti F. (2019). Biodegradation of Plastics in Soil: The Effect of Temperature. Polym. Degrad. Stab..

[23] Endres H.-J., Wagemann K., Tippkötter N. (2019). Bioplastics BT—Biorefineries. Advances in Biochemical Engineering/Biotechnology.

[24] Nakasaki K., Matsuura H., Tanaka H., Sakai T. (2006). Synergy of Two Thermophiles Enables Decomposition of Poly-Ɛ-Caprolactone under Composting Conditions. FEMS Microbiol. Ecol..

[25] Kale G., Kijchavengkul T., Auras R., Rubino M., Selke S.E., Singh S.P. (2007). Compostability of Bioplastic Packaging Materials: An Overview. Macromol. Biosci..

[26] Massardier-Nageotte V., Pestre C., Cruard-Pradet T., Bayard R. (2006). Aerobic and Anaerobic Biodegradability of Polymer Films and Physico-Chemical Characterization. Polym. Degrad. Stab..

[27] Lal R. (2006). Enhancing Crop Yields in the Developing Countries through Restoration of the Soil Organic Carbon Pool in Agricultural Lands. Land Degrad. Dev..

[28] Bünemann E.K., Schwenke G.D., Van Zwieten L. (2006). Impact of Agricultural Inputs on Soil Organisms—A Review. Aust. J. Soil Res..

[29] Gutierrez-Wing M.T., Stevens B.E., Theegala C.S., Negulescu I.I., Rusch K.A. (2010). Anaerobic Biodegradation of Polyhydroxybutyrate in Municipal Sewage Sludge. J. Environ. Eng..

[30] Karamanlioglu M., Robson G.D. (2013). The Influence of Biotic and Abiotic Factors on the Rate of Degradation of Poly(Lactic) Acid (PLA) Coupons Buried in Compost and Soil. Polym. Degrad. Stab..

[31] Luo Y., Lin Z., Guo G. (2019). Biodegradation Assessment of Poly (Lactic Acid) Filled with Functionalized Titania Nanoparticles (PLA/TiO_2_) Under Compost Conditions. Nanoscale Res. Lett..

[32] Borelbach P., Kopitzky R., Dahringer J., Gutmann P. (2023). Degradation Behavior of Biodegradable Man-Made Fibers in Natural Soil and in Compost. Polymers.

[33] Weng Y.-X., Wang X.-L., Wang Y.-Z. (2011). Biodegradation Behavior of PHAs with Different Chemical Structures Under Controlled Composting Conditions. Polym. Test..

[34] Seggiani M., Cinelli P., Verstichel S., Puccini M., Vitolo S., Anguillesi I., Lazzeri A. (2015). Development of Fibres-Reinforced Biodegradable Composites. Chem. Eng. Trans..

[35] Sander M. (2019). Biodegradation of Polymeric Mulch Films in Agricultural Soils: Concepts, Knowledge Gaps, and Future Research Directions. Environ. Sci. Technol..

[36] Kijchavengkul T., Auras R., Rubino M., Selke S., Ngouajio M., Fernandez R.T. (2010). Biodegradation and Hydrolysis Rate of Aliphatic Aromatic Polyester. Polym. Degrad. Stab..

[37] Koitabashi M., Noguchi M.T., Sameshima-Yamashita Y., Hiradate S., Suzuki K., Yoshida S., Watanabe T., Shinozaki Y., Tsushima S., Kitamoto H.K. (2012). Degradation of Biodegradable Plastic Mulch Films in Soil Environment by Phylloplane Fungi Isolated from Gramineous Plants. AMB Express.

[38] Miles C., Wallace R., Wszelaki A., Martin J., Cowan J., Walters T., Inglis D. (2012). Deterioration of Potentially Biodegradable Alternatives to Black Plastic Mulch in Three Tomato Production Regions. HortScience Horts.

[39] Costa P., Pinto F., Miranda M., André R.N., Rodrigues M. (2014). Study of the Experimental Conditions of the Co-Pyrolysis of Rice Husk and Plastic Wastes. Chem. Eng. Trans..

[40] Li G., Li H., Leffelaar P.A., Shen J., Zhang F. (2014). Characterization of Phosphorus in Animal Manures Collected from Three (Dairy, Swine, and Broiler) Farms in China. PLoS ONE.

[41] Selke S., Auras R., Nguyen T.A., Castro Aguirre E., Cheruvathur R., Liu Y. (2015). Evaluation of Biodegradation-Promoting Additives for Plastics. Environ. Sci. Technol..

[42] Moreno M.M., González-Mora S., Villena J., Campos J.A., Moreno C. (2017). Deterioration Pattern of Six Biodegradable, Potentially Low-Environmental Impact Mulches in Field Conditions. J. Environ. Manag..

[43] van der Zee M., Zijlstra M., Kuijpers L.J., Hilhorst M., Molenveld K., Post W. (2024). The Effect of Biodegradable Polymer Blending on the Disintegration Rate of PHBV, PBS and PLA in Soil. Polym. Test..

[44] Sedničková M., Pekařová S., Kucharczyk P., Bočkaj J., Janigová I., Kleinová A., Jochec-Mošková D., Omaníková L., Perďochová D., Koutný M. (2018). Changes of Physical Properties of PLA-Based Blends During Early Stage of Biodegradation in Compost. Int. J. Biol. Macromol..

[45] Stloukal P., Pekařová S., Kalendova A., Mattausch H., Laske S., Holzer C., Chitu L., Bodner S., Maier G., Slouf M. (2015). Kinetics and Mechanism of the Biodegradation of PLA/Clay Nanocomposites during Thermophilic Phase of Composting Process. Waste Manag..

[46] Yagi H., Ninomiya F., Funabashi M., Kunioka M. (2012). Anaerobic Biodegradation of Poly (Lactic Acid) Film in Anaerobic Sludge. J. Polym. Environ..

[47] Dey S., Tribedi P. (2018). Microbial Functional Diversity Plays an Important Role in the Degradation of Polyhydroxybutyrate (PHB) in Soil. 3 Biotech.

[48] Altaee N., El-Hiti G.A., Fahdil A., Sudesh K., Yousif E. (2016). Biodegradation of Different Formulations of Polyhydroxybutyrate Films in Soil. Springerplus.

[49] Swiontek Brzezinska M., Walczak M., Kalwasińska A., Richert A., Świątczak J., Deja-Sikora E., Burkowska-But A. (2020). Biofilm Formation During Biodegradation of Polylactide, Poly (3,4 Hydroxybutyrate) and Poly(ε-Caprolactone) in Activated Sludge. Int. J. Biol. Macromol..

[50] Gutierrez-Wing M.T., Stevens B.E., Theegala C.S., Negulescu I.I., Rusch K.A. (2011). Aerobic Biodegradation of Polyhydroxybutyrate in Compost. Environ. Eng. Sci..

[51] Carta F., Beun J.J., van Loosdrecht M.C.M., Heijnen J.J. (2001). Simultaneous Storage and Degradation of Phb and Glycogen in Activated Sludge Cultures. Water Res..

[52] Dircks K., Henze M., van Loosdrecht M.C.M., Mosbæk H., Aspegren H. (2001). Storage and Degradation of Poly-β-Hydroxybutyrate in Activated Sludge under Aerobic Conditions. Water Res..

[53] Al-Khattaf F.S., Al-Ansari M.M., Maruthamuthu M.K., Dyona L., Agastian P. (2022). Polyhydroxybutyrate Degradation by Biocatalyst of Municipal Sludge Water and Degradation Efficacy in Sequencing Batch Biofilm Reactor. Environ. Res..

[54] Arcos-Hernandez M.V., Laycock B., Pratt S., Donose B.C., Nikolić M.A.L., Luckman P., Werker A., Lant P.A. (2012). Biodegradation in a Soil Environment of Activated Sludge Derived Polyhydroxyalkanoate (PHBV). Polym. Degrad. Stab..

[55] Boonluksiri Y., Prapagdee B., Sombatsompop N. (2021). Promotion of Polylactic Acid Biodegradation by a Combined Addition of PLA-Degrading Bacterium and Nitrogen Source Under Submerged and Soil Burial Conditions. Polym. Degrad. Stab..

[56] Pattanasuttichonlakul W., Sombatsompop N., Prapagdee B. (2018). Accelerating Biodegradation of PLA Using Microbial Consortium from Dairy Wastewater Sludge Combined with PLA-Degrading Bacterium. Int. Biodeterior. Biodegrad..

[57] Gee G.W., Bauder J.W. (1986). Particle-Size Analysis. Methods of Soil Analysis.

[58] McLean E.O. (1982). Soil PH and Lime Requirement. Methods of Soil Analysis, Part 2: Chemical and Microbiological Properties.

[59] Rhoades J.D. (1982). Soluble Salts. Methods of Soil Analysis, Part 2: Chemical and Microbiological Properties.

[60] Bremner J.M., Mulvaney C.S. (1982). Nitrogen—Total. Methods of Soil Analysis, Part 2: Chemical and Microbiological Properties.

[61] Keeney D.R., Nelson D.W. (1982). Nitrogen—Inorganic Forms. Methods of Soil Analysis, Part 2: Chemical and Microbiological Properties.

[62] American Public Health Association, American Water Works Association, World Economic Forum (1998). Standart Methods for the Examination of Water and Wastewater.

[63] Rudnik E., Briassoulis D. Long-Term Biodegradability in Soil of Bio-Based Biodegradable Polymers. Proceedings of the International Conference on Agricultural Engineering-AgEng 2010: Towards Environmental Technologies.

[64] Palsikowski P.A., Kuchnier C.N., Pinheiro I.F., Morales A.R. (2018). Biodegradation in Soil of PLA/PBAT Blends Compatibilized with Chain Extender. J. Polym. Environ..

[65] Briassoulis D., Degli Innocenti F., Malinconico M. (2017). Standards for Soil Biodegradable Plastics BT—Soil Degradable Bioplastics for a Sustainable Modern Agriculture. Soil Degradable Bioplastics for a Sustainable Modern Agriculture.

[66] Akdag Ozkan H.A., Hockenberger A., Cam S.M., Çelen O., Uludoğan C. (2024). Effect of Enhanced Quenching on Properties of Melt Spun Multifilament Poly[3-hydroxybutyrate] Yarns. Text. Res. J..

[67] Chinaglia S., Tosin M., Degli-Innocenti F. (2018). Biodegradation Rate of Biodegradable Plastics at Molecular Level. Polym. Degrad. Stab..

[68] Gürler N., Paşa S., Temel H. (2021). Silane Doped Biodegradable Starch-PLA Bilayer Films for Food Packaging Applications: Mechanical, Thermal, Barrier and Biodegradability Properties. J. Taiwan Inst. Chem. Eng..

[69] Iovino R., Zullo R., Rao M.A., Cassar L., Gianfreda L. (2008). Biodegradation of Poly(Lactic Acid)/Starch/Coir Biocomposites Under Controlled Composting Conditions. Polym. Degrad. Stab..

[70] (2018). ASTM Standard Test Method for Determining Aerobic Biodegradation of Plastic Materials in Soil.

[71] Kale G., Auras R., Singh S.P., Narayan R. (2007). Biodegradability of Polylactide Bottles in Real and Simulated Composting Conditions. Polym. Test..

[72] Wei L., Liang S., McDonald A.G. (2015). Thermophysical Properties and Biodegradation Behavior of Green Composites Made from Polyhydroxybutyrate and Potato Peel Waste Fermentation Residue. Ind. Crops Prod..

[73] Satti S.M., Shah A.A., Marsh T.L., Auras R. (2018). Biodegradation of Poly(Lactic Acid) in Soil Microcosms at Ambient Temperature: Evaluation of Natural Attenuation, Bio-Augmentation and Bio-Stimulation. J. Polym. Environ..

[74] Muniyasamy S., Ofosu O., John M.J., Anandjiwala R.D. (2016). Mineralization of Poly(Lactic Acid) (PLA), Poly(3-Hydroxybutyrate-Co-Valerate) (PHBV) and PLA/PHBV Blend in Compost and Soil Environments. J. Renew. Mater..

[75] Rudnik E., Briassoulis D. (2011). Degradation Behaviour of Poly(Lactic Acid) Films and Fibres in Soil Under Mediterranean Field Conditions and Laboratory Simulations Testing. Ind. Crops Prod..

[76] Tabasi R.Y., Ajji A. (2015). Selective Degradation of Biodegradable Blends in Simulated Laboratory Composting. Polym. Degrad. Stab..

[77] Mihai M., Legros N., Alemdar A. (2014). Formulation-Properties Versatility of Wood Fiber Biocomposites Based on Polylactide and Polylactide/Thermoplastic Starch Blends. Polym. Eng. Sci..

[78] Ren Y., Hu J., Yang M., Weng Y. (2019). Biodegradation Behavior of Poly (Lactic Acid) (PLA), Poly (Butylene Adipate-Co-Terephthalate) (PBAT), and Their Blends Under Digested Sludge Conditions. J. Polym. Environ..

[79] Palucha N., Fojt J., Holátko J., Hammerschmiedt T., Kintl A., Brtnický M., Řezáčová V., De Winterb K., Uitterhaegen E., Kučerík J. (2024). Does Poly-3-Hydroxybutyrate Biodegradation Affect the Quality of Soil Organic Matter?. Chemosphere.

[80] Tokiwa Y., Calabia B.P. (2004). Review Degradation of Microbial Polyesters. Biotechnol. Lett..

[81] Rehman R.A., Rao A.Q., Ahmed Z., Gul A. (2015). Selection of Potent Bacterial Strain for Over-Production of PHB by Using Low Cost Carbon Source for Eco-Friendly Bioplastics. Adv. Life Sci..

[82] Pérez-Arauz A.O., Aguilar-Rabiela A.E., Vargas-Torres A., Rodríguez-Hernández A.-I., Chavarría-Hernández N., Vergara-Porras B., López-Cuellar M.R. (2019). Production and Characterization of Biodegradable Films of a Novel Polyhydroxyalkanoate (PHA) Synthesized from Peanut Oil. Food Packag. Shelf Life.

[83] Kim J., Gupta N.S., Bezek L.B., Linn J., Bejagam K.K., Banerjee S., Dumont J.H., Nam S.Y., Kang H.W., Park C.H. (2023). Biodegradation Studies of Polyhydroxybutyrate and Polyhydroxybutyrate-Co-Polyhydroxyvalerate Films in Soil. Int. J. Mol. Sci..

[84] Volova T.G., Gladyshev M.I., Trusova M.Y., Zhila N.O. (2007). Degradation of Polyhydroxyalkanoates in Eutrophic Reservoir. Polym. Degrad. Stab..

[85] Gasparyan K.G., Tyubaeva P.M., Varyan I.A., Vetcher A.A., Popov A.A. (2023). Assessing the Biodegradability of PHB-Based Materials with Different Surface Areas: A Comparative Study on Soil Exposure of Films and Electrospun Materials. Polymers.

[86] Armentano I., Dottori M., Fortunati E., Mattioli S., Kenny J.M. (2010). Biodegradable Polymer Matrix Nanocomposites for Tissue Engineering: A Review. Polym. Degrad. Stab..

[87] Huda S.M.S., Satoh H., Mino T. (2016). Anaerobic Degradation of Polyhydroxyalkanoate Accumulated in Activated Sludge in the Absence of Anaerobic Digested Sludge. J. Water Environ. Technol..

[88] Walczak M., Swiontek Brzezinska M., Sionkowska A., Michalska M., Jankiewicz U., Deja-Sikora E. (2015). Biofilm Formation on the Surface of Polylactide During Its Biodegradation in Different Environments. Colloids Surfaces B Biointerfaces.

[89] Lee J.C., Moon J.H., Jeong J.-H., Kim M.Y., Kim B.M., Choi M.-C., Kim J.R., Ha C.-S. (2016). Biodegradability of Poly(Lactic Acid) (PLA)/Lactic Acid (LA) Blends Using Anaerobic Digester Sludge. Macromol. Res..

[90] Palai B., Mohanty S., Nayak S.K. (2021). A Comparison on Biodegradation Behaviour of Polylactic Acid (PLA) Based Blown Films by Incorporating Thermoplasticized Starch (TPS) and Poly (Butylene Succinate-Co-Adipate) (PBSA) Biopolymer in Soil. J. Polym. Environ..

[91] Itävaara M., Karjomaa S., Selin J.-F. (2002). Biodegradation of Polylactide in Aerobic and Anaerobic Thermophilic Conditions. Chemosphere.

[92] Martucci J.F., Ruseckaite R.A. (2015). Biodegradation Behavior of Three-Layer Sheets Based on Gelatin and Poly (Lactic Acid) Buried Under Indoor Soil Conditions. Polym. Degrad. Stab..

[93] Boonmee C., Kositanont C., Leejarkpai T. (2016). Degradation of Poly(Lactic Acid) Under Simulated Landfill Conditions. Environ. Nat. Resour. J..

[94] Weng Y.-X., Jin Y.-J., Meng Q.-Y., Wang L., Zhang M., Wang Y.-Z. (2013). Biodegradation Behavior of Poly(Butylene Adipate-Co-Terephthalate) (PBAT), Poly(Lactic Acid) (PLA), and Their Blend Under Soil Conditions. Polym. Test..

[95] Lopez-Llorca L.V., Colom Valiente M.F., Gascon A. (1993). A Study of Biodegradation of Poly-β-Hydroxyalkanoate (PHA) Films in Soil Using Scanning Electron Microscopy. Micron.

[96] Feijoo P., Marín A., Samaniego-Aguilar K., Sánchez-Safont E., Lagarón J.M., Gámez-Pérez J., Cabedo L. (2023). Effect of the Presence of Lignin from Woodflour on the Compostability of PHA-Based Biocomposites: Disintegration, Biodegradation and Microbial Dynamics. Polymers.

[97] Gómez E.F., Michel F.C. (2013). Biodegradability of Conventional and Bio-Based Plastics and Natural Fiber Composites During Composting, Anaerobic Digestion and Long-Term Soil Incubation. Polym. Degrad. Stab..

[98] Corrêa M.C.S., Rezende M.L., Rosa D.S., Agnelli J.A.M., Nascente P.A.P. (2008). Surface Composition and Morphology of Poly(3-Hydroxybutyrate) Exposed to Biodegradation. Polym. Test..

[99] Li L., Frey M., Browning K.J. (2010). Biodegradability Study on Cotton and Polyester Fabrics. J. Eng. Fiber. Fabr..

[100] Gong Q., Wang L.-Q., Tu K. (2006). In Situ Polymerization of Starch with Lactic Acid in Aqueous Solution and the Microstructure Characterization. Carbohydr. Polym..

[101] Sommer K., Van Opdenbosch D., Zollfrank C. (2023). Synthesis and Characterization of Functional Cellulose–Ether-Based PCL- and PLA-Grafts-Copolymers. Polymers.

[102] Cuevas-Carballo Z.B., Duarte-Aranda S., Canché-Escamilla G. (2019). Properties and Biodegradation of Thermoplastic Starch Obtained from Grafted Starches with Poly(Lactic Acid). J. Polym. Environ..

[103] Azeez S., Shenbagaraman R., Ahmed S., Hussain C.M. (2025). 8—Fourier Transform Infrared Spectroscopy in Characterization of Bionanocomposites. Characterization Techniques in Bionanocomposites.

[104] Qin L., Qiu J., Liu M., Ding S., Shao L., Lü S., Zhang G., Zhao Y., Fu X. (2011). Mechanical and Thermal Properties of Poly(Lactic Acid) Composites with Rice Straw Fiber Modified by Poly(Butyl Acrylate). Chem. Eng. J..

[105] Chen C.-C., Chueh J.-Y., Tseng H., Huang H.-M., Lee S.-Y. (2003). Preparation and Characterization of Biodegradable PLA Polymeric Blends. Biomaterials.

[106] Stanley A., Murthy P.S.K., Vijayendra S.V.N. (2020). Characterization of Polyhydroxyalkanoate Produced by Halomonas Venusta KT832796. J. Polym. Environ..

[107] Xiao N., Jiao N. (2011). Formation of Polyhydroxyalkanoate in Aerobic Anoxygenic Phototrophic Bacteria and Its Relationship to Carbon Source and Light Availability. Appl. Environ. Microbiol..

[108] Luo S., Netravali A.N. (2003). A Study of Physical and Mechanical Properties of Poly(Hydroxybutyrate-Co-Hydroxyvalerate) During Composting. Polym. Degrad. Stab..

